# Impact of Endocrine Therapy on Osteoporosis Risk in Women with Breast Cancer Across Different Hormonal Stages: A Review

**DOI:** 10.3390/curroncol32060305

**Published:** 2025-05-26

**Authors:** Beatriz Gomes, Nuno Vale

**Affiliations:** 1 PerMed Research Group, RISE-Health, Faculty of Medicine, University of Porto, Alameda Professor Hernâni Monteiro, 4200-319 Porto, Portugal; pg53482@alunos.uminho.pt; 2 School of Engineering, Universidade do Minho, Campus de Azurém, 4800-058 Guimarães, Portugal; 3 Laboratory of Personalized Medicine, Department of Community Medicine, Health Information and Decision (MEDCIDS), Faculty of Medicine, University of Porto, Rua Doutor Plácido da Costa, 4200-450 Porto, Portugal; 4 RISE-Health, Department of Community Medicine, Health Information and Decision (MEDCIDS), Faculty of Medicine, University of Porto, Rua Doutor Plácido da Costa, 4200-450 Porto, Portugal

**Keywords:** breast cancer, osteoporosis, premenopausal, tamoxifen, rehabilitation, personalized medicine

## Abstract

Breast cancer is the leading cause of death among women, and its treatment often involves chemotherapy and hormone therapy, which can compromise bone mineral density (BMD). Tamoxifen, a selective estrogen receptor modulator, has different effects depending on the patient’s hormonal status. On the one hand, in postmenopausal women, it has a protective effect on BMD; on the other hand, in premenopausal women, it can accelerate bone loss, increasing the risk of osteoporosis and fractures. The reduction in estrogen levels during treatment is a key factor in this bone loss. This review underscores the importance of early risk assessment and regular monitoring of bone mineral density, along with the adoption of individualized pharmacological and non-pharmacological strategies, such as calcium and vitamin D supplementation and physical exercise, to preserve bone health in premenopausal women with breast cancer undergoing endocrine therapy.

## 1. Introduction

Breast cancer (BC) is the second leading cause of death worldwide, ahead of cardiovascular disease [1]. It is characterized by the uncontrolled growth of abnormal cells in the breast tissue, which arise predominantly in the ductal epithelium, giving rise to ductal carcinoma, or in the breast lobules, resulting in lobular carcinoma [2]. In addition, breast cancer can also manifest as triple-negative carcinoma, Paget’s disease of the breast and inflammatory carcinoma, although these variants are less common [3]. Currently, treatment options for breast cancer include surgery, radiotherapy, chemotherapy, hormone therapy, targeted therapy and immunotherapy [4].

The incidence of breast cancer shows a strong correlation with human development. In this way, the Human Development Index (HDI), which combines indicators of life expectancy, education and wealth, has revealed that countries with more developed levels have higher incidences of the disease, although more than half of global cases are diagnosed in low- and middle-income countries, where the impact of the disease on health systems is significant [5]. In 2020, the World Health Organization (WHO) recorded 2,396,840 new cases of breast cancer worldwide [6]. In 2024, in the United States alone, 313,510 new cases and 42,780 deaths were reported due to this pathology [7].

The COVID-19 pandemic in 2020 caused some delays in cancer diagnosis and treatment due to restricted access to health services and fear of exposure to the virus [7]. For this reason, between 2019 and 2021, there was a 46.7% reduction in breast cancer screenings, which resulted in late diagnoses and negatively impacted the survival rate of patients [4].

Osteoporosis is the most common bone disorder in the world and is characterized by a reduction in BMD [8] and deterioration in the microarchitecture of bone tissue, resulting in increased bone fragility and risk of fracture [9]. This pathology is divided into primary and secondary osteoporosis. Primary osteoporosis occurs mainly with aging in women over 50 due to the decrease in estrogen secretion after menopause. Secondary bone disease can occur as a side effect of certain pharmacological treatments that compromise bone homoeostasis. Bone balance depends on a coordinated cycle between bone resorption by osteoclasts and the formation of a bone matrix by osteoblasts, the disruption of which is one of the main pathogenic factors in osteoporosis [8,10].

Breast cancer survivors have significantly low BMD and an increased risk of osteopenia and osteoporosis [11]. Most of the existing studies focus mainly on older postmenopausal patients, with variations in the cancer treatments analyzed [12]. Given the significant impact of osteoporosis on the quality of life of breast cancer survivors and the gaps in the literature regarding premenopausal women, this study aims to investigate the incidence of osteoporosis in breast cancer patients.

### 1.1. Risk Factors Synergy in Breast Cancer and Osteoporosis

The risk factors associated with breast cancer are extensively documented in the literature and encompass both non-modifiable elements, such as age, sex, genetic predisposition, hormonal status, ethnicity, breast density and prior exposure to radiotherapy, and modifiable lifestyle-related factors, including alcohol consumption, poor diet, obesity and smoking [13]. According to a recent report by the World Cancer Research Fund International (WCRF) and the American Institute for Cancer Research (AICR), approximately 29% of cancer cases could have been prevented through the adoption of a healthy lifestyle [14]. It is noteworthy that many of these determinants also play a fundamental role in the pathophysiology of osteoporosis [15]. This overlap in risk factors is of particular clinical relevance, as conditions such as early menopause, aging and sedentary behavior may simultaneously contribute to breast carcinogenesis and the deterioration of bone tissue. In this context, an integrated approach that acknowledges the shared determinants of both pathologies is essential for developing more effective prevention and intervention strategies, focusing on promoting bone health and reducing the oncological risk.

#### 1.1.1. Gender, Age, Tumor Stage and Ethnicity

Breast cancer remains the leading cause of cancer death among women worldwide [16]. In 2024, a significant discrepancy was observed between the genders, with 310,720 new cases diagnosed in women, resulting in 42,250 deaths, while, in men, the figures were considerably lower, with 2790 new cases and 530 deaths [7]. Osteoporosis, another major chronic disease, shows a strong gender bias, being more common in women than in men. According to recent data, the global prevalence of osteoporosis in elderly women is 35.3%, compared to 12.5% in elderly men, confirming a greater female vulnerability to the disease, particularly at an advanced age, due to the marked decline in estrogen levels after menopause [17,18].

Age therefore emerges as a common risk factor across both conditions. In the case of breast cancer, around 84% of cases of invasive breast cancer occur in women aged 50 or over, and approximately 52% of deaths are recorded in women aged 70 or over, demonstrating the relationship between age and mortality. The majority of breast cancer cases are diagnosed at the age of 62 [19]. Similarly, in the case of osteoporosis, there is a significant increase in prevalence with age, with advanced age being a key determinant in the risk of fractures and loss of bone mass [20].

Ethnicity is an important factor in determining the risk of both breast cancer and osteoporosis. In breast cancer, characteristics such as molecular subtype, tumor size and stage at diagnosis vary across ethnic groups. For example, Black women are less likely to be diagnosed with localized disease (58% vs. 68% in White women) and more likely to present with large (≥5 cm) or high-grade tumors. Additionally, the prevalence of triple-negative breast cancer is about twice as high in Black women (19%) compared to other groups (9–11%). These disparities may be linked to genetic, ancestral and social determinants of health, such as poverty and residential segregation [19]. Regarding osteoporosis, significant differences are also observed between ethnic groups, directly impacting the fracture risk. Ethnicity influences bone mineral metabolism [21], and osteoporosis prevalence varies across populations. A study conducted by Wright et al. found that Mexican-American women have a higher risk of osteoporosis compared to White women, who, in turn, are at greater risk than Black women [20].

#### 1.1.2. Hormonal Status and Genetic Factors

Hormonal activity plays a crucial role in the development and function of breast tissue, and an imbalance between estrogen and progesterone may promote the onset of breast cancer. Estrogen, in particular, stimulates cell proliferation by activating hormone receptors and regulating cell cycle pathways [22]. On the other hand, progesterone, also secreted by the ovaries, is essential for regulating the menstrual cycle and differentiating breast tissue. Prolonged exposure to high levels of these hormones, as is the case with combined hormone replacement therapy, can alter this balance and increase the risk of cancer. In addition, reproductive factors such as the early onset of menstruation, late menopause, older age at first childbirth and nulliparity also contribute to this risk, possibly due to prolonged exposure to estrogen. This hormonal imbalance results in excessive activation of hormone receptors and alterations in intracellular signaling, important aspects in the classification of breast cancer subtypes and the choice of personalized therapies [23]. In the context of osteoporosis, low estrogen levels after menopause increase the risk of the disease [18]. Cumulative exposure to endogenous estrogens, reflected in the number of years of menstruation, acts as a protective factor against the development of osteoporosis [24]. Early menopause has also been associated with lower bone mineral density [24] and an increased risk of osteoporosis [25]. Among the key factors associated with the disease, the duration of the postmenopausal period is particularly significant, supporting the view that estrogen deficiency, rather than chronological aging, is the main driver of the disease during this stage of life [26].

In addition to hormonal factors, genetic factors have a considerable impact on breast cancer predisposition, accounting for around 5–10% of cases [27]. Mutations in the BRCA1 and BRCA2 genes are the most widely studied, conferring a high risk of developing breast cancer, with up to an 80% probability [28], particularly in individuals with a family history of the disease. Other genes mutations, such as TP53, PTEN and PALB2, are related to the risk of developing the disease [23]. It is important to note that the frequency of these mutations can vary according to ethnic origin, with a higher prevalence among Jews of Ashkenazi origin. In addition, factors such as consanguinity can increase this risk in certain populations, as observed in studies in Pakistan.

Therefore, genetic testing and specialized counseling are essential to identify individuals at high risk and guide personalized prevention and intervention strategies [29]. Genetic factors also play a central role in osteoporosis, primarily influencing bone mineral density (BMD). Polymorphisms in genes such as VDR (vitamin D receptor), ESR1 (estrogen receptor alpha) and COL1A1 (type I collagen alpha 1) have been consistently associated with low BMD and increased fracture risk by affecting mechanisms such as calcium absorption, hormonal regulation and bone remodeling [30]. Additionally, several single--nucleotide polymorphisms (SNPs) in the ESR1 gene and the major histocompatibility complex (MHC) have been linked to the age of natural menopause and, consequently, to the risk of postmenopausal osteoporosis. A recent study also identified that, in postmenopausal women, the long non-coding RNA SNHG1 was downregulated, with even lower expression levels observed in women with osteoporosis, suggesting its potential as a biomarker for postmenopausal osteoporosis [18]. Other genes related to inflammatory cytokines (such as IL-6 and IL-1ra) and bone metabolism (such as IGF-I and TGF-β1) also contribute to genetic susceptibility, particularly after menopause [30].

#### 1.1.3. Benign Breast Changes

Breast density is a risk factor for the development of breast cancer, especially in women with highly dense breast tissue [2]. Mammographic density is determined by the proportion of fibroglandular tissue in relation to adipose tissue. In this way, women with higher breast density have more difficulties in detecting tumors early on, since they can be masked on mammograms, resulting in later diagnoses [31]. When converting these data to absolute lifetime risk, it was observed that women with lower breast density have a 6.2% chance of developing breast cancer, while those with high density have a 14.7% risk. These data reinforce the association between high breast density and an increased lifetime risk of developing the disease [32]. In addition to the oncological risk, the association between breast density and estrogen hormone levels suggests a possible link between mammographic density and BMD [30]. Considering that estrogens exert a protective effect on BMD, it is plausible that women with high breast density may have a lower predisposition to osteoporosis during the premenopausal period. However, with menopause and the consequent sharp decline in estrogen levels, this profile may change, making these women more vulnerable to accelerated bone loss.

#### 1.1.4. Previous Treatment Using Radiation Therapy

In addition to the factors intrinsic to the biology of the tumor and the host, treatments play a crucial role in the fight against cancer. Radiotherapy, in particular, is fundamental in the treatment of breast cancer, making a significant contribution to preventing local recurrences and improving long-term survival. This treatment uses high-energy radiation or waves, such as X-rays, gamma rays, electrons or protons, to destroy or prevent cancer cells from multiplying [33].

However, radiotherapy can have long-term side effects, notably cardiac toxicity and damage to bone tissue. In cases of tumors located in the left breast, the heart is more exposed to radiation, increasing the risk of coronary artery disease [29,34]. A study conducted by Carlson et al. [34] reinforced this evidence by showing that women between the ages of 25 and 54 treated with radiotherapy are more likely to develop coronary artery disease than those treated on the right side. Furthermore, radiotherapy can significantly compromise bone health, promoting the development of osteoporosis. Radiation exposure affects the balance between bone formation and resorption by causing dysfunction and death of osteocytes and inhibiting the activity of osteoblasts and osteoclasts, which are essential for the bone remodeling process [35,36].

#### 1.1.5. Health Behaviors: Alcohol, Diet, Obesity and Smoking

Alcohol consumption, poor diet and smoking act synergistically, exacerbating hormonal, inflammatory and metabolic changes that contribute not only to an increased risk of developing and progressing breast cancer but also to a higher risk of osteoporosis.

Alcohol consumption has been associated with increased estrogen levels, the induction of oxidative stress and DNA damage, significantly contributing to the risk of breast cancer [37], with just one alcoholic drink per day potentially increasing this relative risk by approximately 10% [38]. Excessive intake, more than 35 g per day, further increases the risk, particularly for ductal and lobular tumors, with relative risks reaching up to 1.52 times higher [39]. In osteoporosis, chronic and excessive alcohol consumption significantly compromises bone health by reducing bone mineral density and weakening the mechanical properties of bone, thereby increasing the risk of developing the disease. On the other hand, the effects of moderate alcohol consumption on bone health remain less clear [40,41].

Diet can significantly influence the risk of breast cancer, although the data remain inconclusive. Diets high in saturated fats, cholesterol and red and processed meats, as well as excessive egg consumption, have been associated with an increased risk of the disease. In contrast, certain foods and dietary patterns, such as dairy products (especially yogurt), low-fat products, moderate folate intake, soy consumption (notably among Asian women), n-3 fatty acids found in fish and possibly the Mediterranean diet, appear to have a protective effect. Tea and coffee consumption does not show a clear association, although caffeine may benefit women with ER-negative tumors. Further studies are needed to confirm these associations [42,43].

Beyond its influence on cancer risk, diet also plays a key role in bone health. An adequate intake of calcium and vitamin D contributes to the maintenance of bone mineral density and is considered protective against osteoporosis. Conversely, nutritional deficiencies, particularly in older adults or individuals with a lower genetic predisposition for osteoporosis, may accelerate bone loss, thereby increasing the risk of fractures and other related complications [41,44]. Thus, a balanced diet is crucial for the simultaneous prevention of both breast cancer and osteoporosis [45]. Obesity is another important risk factor, especially in postmenopausal women. Studies indicate that obese or overweight women have a lower breast cancer survival rate (55.6%) compared to normal weight women (79.9%), as well as a higher frequency of bulky and lymph node-invasive tumors [42]. It is therefore recommended that postmenopausal women adopt healthy habits, such as a balanced diet and regular physical activity, in order to reduce the risks associated with excess weight [14]. Similarly, although some individuals present with normal or above-average bone mineral density (BMD), they exhibit high fracture rates, suggesting poor bone quality [46].

Smoking, known for its carcinogenic effects and for promoting lung metastases in patients with breast cancer [47], is significantly associated with an 18% [48] increase in the risk of lung metastases in women with invasive breast cancer, as well as a 33% reduction in survival rate at the time of diagnosis [49]. Nicotine may promote tumor progression by interfering with the immune response in the lungs in addition to negatively affecting bone health. In animal models, nicotine administration resulted in an increased number of osteoclasts and a reduction in bone mineral density (BMD), indicating an imbalance in bone remodeling that favors bone mass loss [50]. Thus, quitting smoking after a breast cancer diagnosis has proven benefits, significantly improving survival, which reinforces the importance of effective smoking cessation strategies [51].

## 2. Breast Cancer and Osteoporosis: Relationship and Impacts

### 2.1. Breast Cancer Classification and Its Therapeutic Implications

Breast cancer is a heterogeneous pathology that requires specific therapeutic strategies adapted to the biological characteristics of each tumor and the clinical stage of the disease. The most common subtype is carcinoma, which develops from the epithelial cells lining the breast ducts or lobules [52]. Histologically, it is classified as either carcinoma in situ (ductal carcinoma in situ or lobular carcinoma in situ), when the tumor remains confined to its site of origin, or invasive carcinoma (invasive ductal or lobular carcinoma), when the cancer breaches the epithelial layer and invades adjacent tissues, increasing the risk of metastatic spread [3,53].

In addition to histological classification, molecular profiling has become a cornerstone of personalized breast cancer treatment. This classification identifies subtypes such as Luminal A, Luminal B, HER2-enriched and Basal-like (triple-negative), based on the expression of hormone receptors (estrogen and progesterone) and the HER2 protein [53]. This stratification has transformed clinical practice, enabling more precise and effective therapeutic approaches tailored to the biological characteristics of each tumor.

In simplified terms, tumors can be grouped into four major molecular categories: HR+/HER2-, HR+/HER2+, HR-/HER2+ and triple-negative (TNBC) [54]. Each of these subtypes has distinct therapeutic implications. HR+/HER2- tumors, the most common, respond well to endocrine therapy, such as tamoxifen or aromatase inhibitors, depending on the patient’s menopausal status [55]. HR+/HER2+ tumors benefit from a combination of hormonal therapy and anti-HER2 agents, such as trastuzumab [56,57]. HR-/HER2+ tumors require chemotherapy combined with HER2-targeted therapies. Conversely, triple-negative tumors, due to the lack of therapeutic targets, are treated mainly with conventional chemotherapy [58].

In the case of early-stage disease (stages I–III), the tumor can be removed by mastectomy or conservative surgery, such as lumpectomy, always accompanied by axillary staging to assess the disease spread. To minimize the risk of local recurrence, radiotherapy is often recommended, especially after conservative surgery or when there is lymph node involvement [59]. In addition to local therapy, systemic treatment, which may include chemotherapy, targeted therapies and hormonal therapy, plays a crucial role in preventing relapse and improving survival.

Approximately 70–75% of breast cancers express positive hormone receptors (HR+), making them susceptible to hormone therapy [60,61]. This approach aims to inhibit tumor growth by interfering with the action of estrogens, either by blocking hormone receptors in tumor cells or by reducing the endogenous production of these hormones [62]. It is one of the pillars of systemic treatment in HR+ patients and is adapted according to the patient’s hormonal status.

In premenopausal women, whose primary source of estrogen is ovarian, endocrine therapy may consist of tamoxifen alone or in combination with ovarian suppression using gonadotropin-releasing hormone (GnRH) agonists [60]. Ovarian suppression induces a hormonal state similar to postmenopause, thereby enhancing the antiproliferative effect of tamoxifen. In postmenopausal women, the main source of estrogen is the peripheral conversion of androgens in adipose tissue. In this context, aromatase inhibitors (AIs) have become the preferred approach, demonstrating greater efficacy than tamoxifen in improving overall survival, particularly in metastatic disease [61,63,64]. AIs are indicated in various clinical contexts, from early-stage disease to local progression or metastatic settings, and are also used to reduce the risk of recurrence following surgery or other medical interventions [61]. Nevertheless, tamoxifen remains a valid option in selected cases and can be administered for up to ten years, based on an individual risk–benefit assessment [65].

### 2.2. Impact of Cancer Treatments on Bone Health

Despite their proven oncological benefits, endocrine therapy can significantly impact bone health, especially in younger patients. Tamoxifen, while functioning as an estrogen receptor antagonist in breast tissue, exerts tissue-specific effects, including variable actions on bone [66]. Furthermore, ovarian suppression, whether surgical or through the use of gonadotropin-releasing hormone (GnRH) agonists, leads to a marked drop in estrogen levels, mimicking premature menopause [67]. This abrupt hormonal change disrupts bone metabolism, enhances bone resorption and reduces bone mineral density (BMD), thereby increasing the risk of osteoporosis and fractures [59,68,69]. Consequently, the link between breast cancer and bone loss is largely attributable to the side effects of treatment rather than the malignancy itself. It is estimated that up to 80% of breast cancer survivors experience long-term BMD reduction, making bone preservation a critical component of survivorship care [70].

Among the breast cancer treatments that affect bone health, hormone therapy such as aromatase inhibitors (AIs) and chemotherapy are particularly impactful [69]. In premenopausal women with breast cancer, these therapies, especially when involving gonadotropin-releasing hormone (GnRH) analogs and tamoxifen, can induce substantial bone loss [71]. Chemotherapy regimes such as cyclophosphamide, anthracyclines, taxanes and platinum compounds are strongly linked to chemotherapy-induced ovarian failure (CIOF), with often manifests as amenorrhea within six months of therapy initiation [59]. Approximately 70% of premenopausal women develop chemotherapy-induced amenorrhea and/or premature menopause, which increases the risk of developing osteoporosis and fractures [72]. When chemotherapy is administered after surgery, ovarian insufficiency develops within 1 year of treatment in 63–96% of premenopausal women [71]. The reduction in estrogen production, whether due to chemotherapy-induced ovarian suppression or inhibition of the aromatase enzyme, compromises the balance between bone renewal and resorption, resulting in a continuous decrease in bone density throughout the therapeutic period, which can vary between 5 and 10 years. This effect is enhanced by additive risk factors, such as prolonged exposure to glucocorticoids during treatment, further increasing the risk of osteoporosis and fractures in young women under 45 undergoing endocrine therapies [59,73].

GnRH analogs, such as goserelin, are commonly employed as part of endocrine therapy in premenopausal women. These long-acting agents induce a hypoestrogenic state by desensitizing pituitary GnRH receptors and suppressing luteinizing hormone secretion, thereby leading to ovarian insufficiency. Within the first six months of treatment, over 95% of patients develop amenorrhea, and the ensuing estrogen deficiency precipitates loss of both cortical and trabecular bone mass [71].

#### Tamoxifen

Since the 1970s, tamoxifen has been widely used as adjuvant and neoadjuvant endocrine therapy for the treatment of estrogen receptor-positive (ER+) breast cancer in both premenopausal and postmenopausal women [74,75,76], demonstrating a significant reduction in the risk of recurrence and mortality [76]. Its efficacy is well documented, showing that five years of tamoxifen reduces the risk of recurrence by approximately 40% and breast cancer-specific mortality by about one-third in HR+ patients [77,78]. Extended use up to ten years provides additional survival benefits compared to five years of treatment [79]. Approved by the Food and Drug Administration (FDA), it is used by both women and men, not only for the treatment of invasive disease but also as adjuvant therapy after surgery and radiotherapy, in the treatment of ductal carcinoma in situ to prevent invasive cancer and even to reduce the risk of breast cancer in high-risk patients [80]. Classified as a selective estrogen receptor modulator (SERM), tamoxifen acts as an antagonist in breast tissue, inhibiting tumor growth, while exerting agonist effects in other tissues such as bone, blood vessels and the uterus. This dual action requires careful monitoring of its long-term effects, particularly on bone tissue, which response varies depending on the woman’s hormonal status [76].

**Figure 1:**
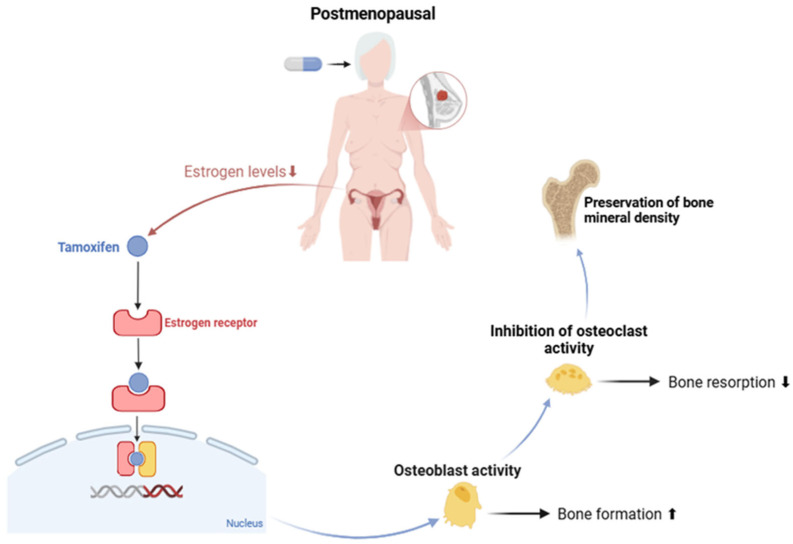
Effect of tamoxifen on estrogen regulation and bone health in postmenopausal women. Created with BioRender.com. Available online: https://www.biorender.com/ (accessed on 24 February 2025).

As previously mentioned, after menopause, the ovaries stop producing estrogen, which leads to a decline in its levels and thus compromises the bone balance [81]. However, in postmenopausal women taking tamoxifen, this is not the case, as this drug has a protective effect. References [82,83,84,85,86,87,88] showed that tamoxifen binds to estrogen receptors in bone, acting as a partial agonist. Due to low competition with natural estrogen, tamoxifen is able to partially mimic its actions on osteoblasts, stimulating their activity and promoting bone formation. In addition, it inhibits the actions of osteoclasts, reducing bone resorption. As a result, BMD is preserved, reducing the risk of osteoporosis and fractures in postmenopausal women. This mechanism is shown in Figure 1. Most clinical trials on the impact of tamoxifen on BMD have focused predominantly on this population, while the adverse effects on premenopausal women remain poorly explored [76].

**Figure 2:**
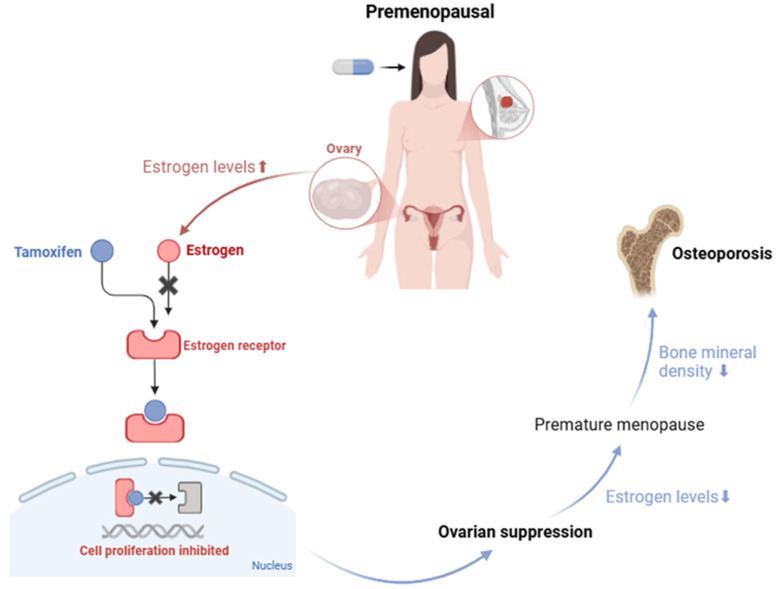
Effect of tamoxifen on estrogen regulation and bone health in premenopausal women. Created with BioRender.com. Available online: https://www.biorender.com/ (accessed on 24 February 2025).

There is a substantial gap in the literature regarding the impact of tamoxifen on BMD in premenopausal women, as most clinical trials focus on postmenopausal women, leaving the adverse effects in this population relatively unexplored [76]. Nevertheless, it is known that premenopausal women have significantly elevated serum estrogen levels [12] and that tamoxifen, by binding to estrogen receptors in breast tissue, blocks the action of endogenous estrogen, thereby inhibiting cell proliferation [72,81,82,83,84,85,86,87,89]. In response to the blockade of estrogen receptors, there is an increase in ovarian estrogen production. However, when tamoxifen is administered in combination with chemotherapy or ovarian suppression therapies, the estrogen levels can drop dramatically, inducing early menopause [67]. This sharp decline in estrogen compromises the maintenance of BMD [59], thereby increasing the risk of osteoporosis and fractures [59,68,69,90]. This mechanism is illustrated in Figure 2.

A study by Powles et al. [82] illustrated this duality. In premenopausal women, tamoxifen caused a progressive decrease in BMD in the lumbar spine (*p* < 0.001) and hip (*p* < 0.05), with an average annual loss of 1.44% in lumbar BMD. In comparison, the placebo group showed a small gain of 0.24% over three years (*p* < 0.001). In postmenopausal women, tamoxifen showed an average annual increase of 1.17% in the BMD of the lumbar spine (*p* < 0.005) and 1.71% in the hip (*p* < 0.001), while the placebo had no significant impact on BMD. These results indicate that tamoxifen may have opposite effects depending on the hormonal phase, being protective for postmenopausal women but detrimental for premenopausal women [91,92].

In addition, Kyvernitakis et al. [91] reported that the risk of fractures in premenopausal patients treated with tamoxifen was 75% higher than the healthy control groups. Among patients aged between 18 and 50, the cumulative incidence of fractures was 6.3% in the tamoxifen-treated group, compared to 3.6% in the control group. However, some studies suggest that tamoxifen is not associated with an increased risk of osteoporosis or osteoporotic fractures in premenopausal breast cancer patients [93]. Although tamoxifen exerts antiestrogenic effects in breast tissue, its partial estrogen agonist activity in bone suggests that, by itself, it may not be intrinsically harmful to bone tissue. Therefore, the negative effects on bone mineral density (BMD) observed in premenopausal women are more likely related to concomitant chemotherapy or ovarian suppression therapies, rather than to tamoxifen alone [94]. In the absence of ovarian suppression, endogenous estrogen production is maintained, and tamoxifen acts as a partial agonist in bone tissue. In this context, tamoxifen partially “replaces” natural estrogen, exerting an insufficient protective effect, which may lead to a slight reduction in bone mineral density in the long term [95]. Nevertheless, these previous studies have certain limitations. The sample sizes were small, and only univariate analyses were conducted to compare differences between the tamoxifen and placebo groups. Additionally, the primary outcome was the percentage change in bone mineral density (BMD), rather than clinically meaningful endpoints such as osteoporosis or osteoporotic fractures, and the follow-up periods were short. For these reasons, the effect of tamoxifen on osteoporosis risk in premenopausal breast cancer patients remains controversial [93]. Therefore, personalized screening strategies are needed for breast cancer survivors with varying risk profiles for osteoporosis [93].

This complexity of actions and side effects reinforces the need for further research, especially to understand how tamoxifen impacts bone health in young women and how prevention strategies can be implemented to mitigate these risks.

## 3. Therapeutic Approaches and Rehabilitation

Osteoporosis is a pathological condition that can cause pain, fractures and physical disability, requiring long-term medical follow-up [96]. In BC patients, preserving bone health requires a personalized therapeutic approach that combines pharmacological interventions and non-pharmacological strategies. Appropriate treatment should include the periodic monitoring of BMD through tests such as DEXA, the use of oral or intravenous bone-modifying agents, calcium and vitamin D supplementation and physical activity, with an emphasis on weight-bearing exercises [59,88,97].

### 3.1. Diagnosis and Risk Assessment

Early diagnosis and ongoing assessment of the risk of osteoporosis are essential for successful rehabilitation in breast cancer patients, especially in pre- and postmenopausal women. Although there is still no consensus on the diagnosis and treatment of osteoporosis in premenopausal women due to the few studies carried out on this population [98], it is known that the BMD value and the risk of fractures should be monitored constantly, since, without screening, the loss of bone mass can go unnoticed until fractures or skeletal deformities occur [81]. Several methods are currently available to evaluate the risk of osteoporotic fractures, with two of the most commonly used being the Fracture Risk Assessment Tool (FRAX) and dual-energy X-ray absorptiometry (DXA). FRAX, created by the World Health Organization (WHO), is a digital algorithm that estimates an individual’s 10-year probability of sustaining a fracture related to osteoporosis. The assessment takes into account a combination of clinical variables, including demographic factors (such as age, sex and BMI); lifestyle habits (e.g., smoking and alcohol consumption); personal and family fracture history; underlying medical conditions like rheumatoid arthritis; the use of glucocorticoids and, when available, bone mineral density (BMD) at the femoral neck [59]. DXA, on the other hand, is a non-invasive and low-radiation imaging procedure that remains the gold standard for measuring BMD. It operates by using two X-ray beams at different energy levels to distinguish between bone and surrounding soft tissues, producing a precise estimation of bone density. The lumbar spine and proximal femur are the primary anatomical sites evaluated. In cases where DXA is not accessible, heel quantitative ultrasound may be considered a feasible alternative for preliminary bone health assessment [59].

The WHO classification for premenopausal women with osteoporosis, based on the T-Score, should not be used, since these women generally have higher bone density and therefore a higher T-score, which does not adequately reflect the risk of osteoporosis given that the incidence of fractures in this population is low, unlike what happens in postmenopausal women [99,100,101]. In these cases, the interpretation of BMD results should be based on the z-score, as shown in Table 1.

Even so, long-term randomized controlled clinical trials are needed in the case of premenopausal women taking tamoxifen, although the low incidence of fractures in this population continues to make it difficult to accurately assess its anti-fracture benefits [86,99,100].

According to the American Society of Clinical Oncology (AS-CO) guidelines, patients with non-metastatic disease taking a cancer treatment that increases the risk of bone loss should undergo BMD examinations, using DXA, every 2 years and more frequently depending on the severity and rate of decline [59,72]. Continuous assessment and the appropriate use of these diagnostic tools make it possible to identify osteoporosis early, reduce the risk of fractures and improve the quality of life of breast cancer patients.

Studies indicate that there are variations in fracture risk between different ethnicities. Barrett-Connor et al. [102] concluded that the absolute risk of fracture shows significant differences between ethnic groups and suggested that specific clinical guidelines should be adopted for each ethnic group. A population-based study by Cauley et al. [103] revealed that the rate of hip BMD loss is, on average, twice as high in Caucasian women as in African-American women, with an increase in this loss as age advances in both groups. The identification of the hormonal and biochemical factors responsible for these ethnic differences and for the increase in bone loss with advancing age needs to be identified in order to ensure more effective and personalized treatment [103].

### 3.2. Therapeutic Approaches to Preventing Osteoporosis

The treatment of osteoporosis in women with a history of breast cancer should be carefully adjusted, considering the effects of drugs on bone mineral density and the risk of fractures [104]. For premenopausal women undergoing treatment for breast cancer or long-term use of glucocorticoids, pharmacological therapy may be necessary to prevent excessive bone loss and the risk of fractures.

Treatment options include antiresorptive drugs such as estrogen and bisphosphonates or anabolic agents such as teriparatide, as shown in Table 2. However, the use of SERMs, such as raloxifene, are not recommended for women who are still menstruating, as they block the action of estrogen and can aggravate bone loss [82,90]. Bisphosphonates are considered the first-line therapy for premenopausal women with primary osteoporosis [105,106], although they are not recommended for women with fertility aspirations [107]. Alendronate has proven to be effective in preventing bone loss associated with the use of glucocorticoids [108], while denosumab has shown benefits in increasing bone mineral density and preventing fractures in women with severe osteoporosis [109]. Studies reinforce the efficacy of bisphosphonates in protecting against chemotherapy-induced bone loss. Delmas et al. [110] evaluated 53 women aged between 36 and 55 with breast cancer and early menopause induced by chemotherapy, divided into two groups: one received risedronate and the other a placebo, both stratified by previous tamoxifen use. After two years, risedronate significantly increased BMD in the lumbar spine and hip, while the placebo group showed a reduction in BMD. Stopping treatment led to bone loss, suggesting the need for continuous use of the drug to maintain its benefits. Another study, conducted by Fuleihan et al. [111], evaluated the efficacy of pamidronate in preventing bone loss in 40 premenopausal women with newly diagnosed breast cancer who had undergone chemotherapy. In a one-year randomized, placebo-controlled trial, more than half of the patient’s developed amenorrhea, which was associated with greater bone loss. Pamidronate prevented this loss in the lumbar spine and hip, with an average difference of 5.0–5.2% in BMD compared to the placebo. In addition, the treatment was well tolerated, suggesting that pamidronate may be an effective strategy for protecting the bone health of these patients.

Finally, although bisphosphonates, teriparatide, denosumab and treatment with estrogens increase bone density in premenopausal women with osteoporosis, there is still no conclusive data confirming the short-term prevention of fractures with the use of these agents [106]. Therefore, the best therapeutic approach should be chosen based on the patient’s clinical profile and the risk–benefit ratio of each treatment.

### 3.3. Non-Pharmacological Therapy

Exercise and muscle strengthening offer a number of health benefits. Although weight-bearing exercise may only result in modest increases in bone density, the improvements in agility, strength and balance provided by regular weight-bearing exercise and muscle strengthening can significantly reduce the risk of falls and subsequent fractures, independently of an increase in bone density [112]. A meta-analytical study conducted by Kelley et al. [113] confirmed the beneficial effects of impact exercise on the bone mineral density of the femoral neck and lumbar spine in premenopausal women. Similarly, Fornusek et al. [114] suggested that regular exercise can contribute to clinically relevant health preservation in patients with the same pathology mentioned above.

A balanced diet plays a crucial role in promoting bone health. To maintain optimal bone mass, it is essential to ensure an adequate intake of vitamins, minerals and proteins. Adequate calcium intake is essential for maintaining bone health. For premenopausal women, the generally recommended daily intake of calcium is 1000 mg. At the same time, vitamin D plays an essential role in calcium absorption [112], and its recommended daily dose ranges from 800 to 1000 IU [9,72]. It is advisable to check the levels of these nutrients before starting treatments that could cause bone loss, especially in patients identified as having low bone mass or osteoporosis on DXA scans [9].

Smoking is also associated with reduced bone density, so it is essential to encourage all patients to give up smoking. In addition, excessive alcohol consumption can also compromise bone health. Patients with osteoporosis should be advised to avoid alcohol abuse, as drinking just two alcoholic drinks a day has been associated with an increased risk of fractures [112].

## 4. Future Perspectives in Personalized Medicine

Personalized medicine has the potential to revolutionize cancer treatment by adapting it to each patient’s genetic and biochemical factors and lifestyle. The aim of this approach is to optimize the tumor response, minimizing side effects and improving the patient’s quality of life [115]. Understanding the mechanisms underlying individual conditions is crucial to developing personalized strategies aimed at correcting nutritional deficiencies and optimizing physiological responses in specific populations. From a clinical perspective, personalized interventions will not only increase treatment efficacy but also improve patient compliance. Interventions based on metabolic, inflammatory and hormonal biomarkers will allow for dynamic and flexible adjustments, adapting to physiological changes, such as those associated with premenopause. Although integrating these approaches into public health systems, especially in settings with limited resources, is a challenge, precision medicine promises a deeper understanding of human health. This will enable targeted treatments for chronic diseases and, in time, truly individualized healthcare, especially for vulnerable populations [116].

Currently, there is a significant gap in studies exploring the mechanisms by which tamoxifen affects bone metabolism in premenopausal women and how these effects vary according to the hormonal and clinical profiles of each patient. Furthermore, there is a lack of robust comparative research that contrasts this population taking tamoxifen with control groups, such as healthy women, women on a placebo or women undergoing chemotherapy alone [77]. Prospective randomized clinical trials in this area are essential to clarify the impact of tamoxifen on BMD and the risk of osteoporosis, as well as assessing its long-term adverse effects. These data are fundamental for the development of personalized therapies that minimize bone loss in breast cancer survivors, ensuring that endocrine therapy, although vital, does not compromise patients’ bone health and quality of life.

Osteoporosis in breast cancer survivors represents a significant challenge, affecting physical health, mobility and quality of life. The loss of BMD and increased risk of fractures can limit independence and emotional well-being. Therefore, rehabilitation should focus on fracture prevention, with personalized exercise programs that include resistance training and weight-bearing activities, as well as nutritional interventions such as calcium and vitamin D supplementation. Education about healthy habits, such as avoiding smoking and moderating alcohol consumption, is also essential. These strategies aim not only to preserve bone health but also to improve quality of life, promoting autonomy and social reintegration.

In this way, personalized medicine can revolutionize the management of osteoporosis in women by adapting treatment strategies to the needs of each patient. The integration of risk assessment tools, genetic profiles, lifestyle adjustments, imaging technologies and targeted pharmacological interventions can optimize treatment outcomes and reduce the impact of osteoporosis-related fractures, especially as an adverse effect of breast cancer treatment in premenopausal women. Despite the challenges, such as the need for access to comprehensive health data and overcoming ethical concerns, new studies and technological advances could advance the sector, allowing for a more personalized and effective approach, improving patients’ quality of life and ensuring that the fight against cancer does not compromise bone structure and mobility in the long term [117].

## Figures and Tables

**Table 1:** Bone mineral density (BMD) classification in premenopausal women based on z-score.

Category	Z-Score	Meaning
Normal	z-score ≥ −2.0	Bone density is within the expected range for age, sex, and ethnic group.
Low BMD	z-score < −2.0	Bone density is lower than expected for age, sex, and ethnic group.

**Table 2:** Drugs used in the treatment of osteoporosis.

Pharmacological Class	Drug	References
Bisphosphonate (Antiresorptive agent)	Alendronate	[105,106,108,110,111]
	Risedronate	
	Pamidronate	
Anabolic agent (Parathyroid hormone analog)	Teriparatide	[106]
Monoclonal antibody against RANKL (Antiresorptive)	Denosumab	[109]
Hormonal therapy (Antiresorptive/Anabolic effect)	Estrogens	[106]

## Abbreviations

BMD	Bone Mineral Density
BC	Breast Cancer
WHO	World Health Organization
SERM	Selective Estrogen Receptor Modulator
FRAX	Fracture Risk Assessment Tool
DXA	Dual-Energy X-ray Absorptiometry
